# Supplementary material for: Germination at Extreme Temperatures: Implications for Alpine Shrub Encroachment
Source: Plants (Basel). 2021 Feb 9;10(2):327. doi: 10.3390/plants10020327 (PMC7915672; doi:10.3390/plants10020327)
Supplement: Supplementary file 1 [file plants-10-00327-s001.pdf]

Table S1. Seed collection information for the species used in this study including the families, species names and their stored seed bank source, seed accession number, location description and habitat notes at the time of collection if available. VCSB denotes Victorian Conservation Seed Bank, Royal Botanic Gardens Victoria, APB denotes Australian PlantBank, Australian Botanic Garden Mount Annan.

Family	Species	Seed source / collection year	Accession Number	Location description	Habitat notes if available	Decimal Latitude	Decimal Longitude
Asteraceae	*Ozothamnus alpinus*	VCSB 2016	2325703	VIC, Alpine National Park. Bogong High Plains. Near the summit of Mt McKay.	Substrate: brown gravelly loam. Alpine herbfield. Associated species include: *Kunzea muelleri, Grevillea australis, Orites lancifolia, Melicytus sp aff dentatus, Olearia frostii, Brachyscome rigidula, Celmisia pugioniformis, Microceris lanceolata, Aciphylla glacialis, Podocarpus lawrencei, Craspedia sp.*	−36.8767	147.2408
Asteraceae	*Olearia algida*	APB 2011	20110245	NSW, Southern Tablelands, Kosciuszko National Park, C. 150 metres along service trail to Mt Kosciuszko from Charlotte Pass car park.		−36.432111	148.327944
Ericaceae	*Epacris glacialis*	APB 2010	20100182	NSW, Southern Tablelands, Kosciuszko National Park, East side of road,1.6 km above Spencers Creek on the Kosciuszko Road towards Charlotte Pass		−36.429806	148.344167
Ericaceae	*Epacris paludosa*	APB 2011	20110080	NSW, Central Tablelands, Kanangra Boyd National Park, C. 100 metres downstream (west) from road crossing of Boyd River (aka Morong Creek)		−33.970278	150.05575
Ericaceae	*Epacris petrophila*	VCSB 2008	2296687	VIC, Yarra Ranges National Park. At Echo Flat Lake Mountain.	Substrate: brown peaty loam. Moist alpine heath. Associated	−37.4883	145.8819

					species include: *Leionema phylicifolia, Prostanthera cuneata, Viola betonicifolia, Ozothamnus sp. 1, Olearia algida, Asterolasia trymalioides, Baeckea gunniana,* various grasses and sedges.		
Ericaceae	*Pentachondra pumila*	VCSB 2013	2364003	VIC, Alpine National Park. Bogong High Plains. Beside walking track to Cope Creek about 400 metres from the Bogong High Plains Road.	Alpine herbfield on brown clay.	−36.9075	147.287778
Ericaceae	*Richea continentis*	VCSB 2015	2384931	Mount Baw Baw, Phillack Saddle.	Open Snow Gum woodland with *Richea* dominated understorey.	−37.82916	146.291667
Fabaceae	*Hovea montana*	VCSB 2007	2296422	VIC, Lake Mountain Alpine Reserve. At Echo Flat about 1 km from Gerratys car park.	Substrate: dark brown peaty soil. Growing in Snow Gum woodland amongst tall heath. Associated species: *Eucalyptus pauciflora, Ozothamnus sp. 1, Leionema phylicifolium, Olearia algida, Orites lancifolia, Viola betonicifolia ssp betonicifolia, Scaevola hookeri, Prostanthera cuneata.*	−37.49	145.8819
Fabaceae	*Oxylobium ellipticum*	VCSB 2016	2388738	VIC, Alpine National Park, Neilsons Crag (The Watchtower)	Subalpine rocky shrubland/mallee scrub.	−37.36166	146.84
Lamiaceae	*Prostanthera cuneata*	VCSB 2006	2280907	VIC, Echo Flat, Lake Mountain about 1 km from Gerattys car park.	Alluvial sandy loam. Dense subalpine tall heathland. Associated species: *Eucalyptus pauciflora, Leionema phylicifolium, Ozothamnus secundiflorus, Podolobium alpestre, Orites lancifolia, Ozothamnus sp. 1,*	−37.4914	145.8803

					Leptinella filicula, Viola betonicifolia ssp betonicifolia, Stylidium armeria, Richea continentis.		
Myrtaceae	*Kunzea muelleri*	APB 2011	20110101	NSW, Southern Tablelands, Kosciuszko National Park, C.2 km east of Happy Jack's Plain on Happy Jack's Road.		−36.051083	148.521667
Thymaleaceae	*Pimelea ligustrina* subsp. *ciliata*	APB 1986	860694	TAS, Central Highlands, 5.4 km north of Guildford Junction on Murchison Highway.		−41.4	145.583333
Violaceae	*Melicytus dentatus*	APB 2007	20070071	NSW, Southern Tablelands, C. 600 m west of Old Adaminaby turnoff on Snowy Mountains Highway.		−35.998611	148.746944
Winteraceae	*Tasmannia xerophila* subsp. *xerophila*	VCSB 2006	2280911	VIC, Blue Range Rd at the Storm Creek crossing. About 10 km NNE of Marysville.	Brown mountain loam. Tall mountain forest. Associated species: *Eucalyptus delegatensis, Nothofagus cunninghamii, Acacia obliquinervia, Leptospermum grandifolium, Senecio gunnii, Derwentia derwentiana ssp derwentiana, Ozothamnus secundiflorus, Pultenaea muelleri, Polystichum proliferum, Stylidium armeria, Viola eminens.*	−37.4378	145.8047
